# Treatment of Nervous Constipation

**Published:** 1846-12

**Authors:** 


*Treatment of Nervous Constipation.*—Prof. Lippich combats constipation, when it obstinately resists the means ordinarily employed, by the use of the following lavement:

| | | |
|---|---|---|
| ℞ | Asafœtida, - - - - - - - | 12 grammes. |
| | Common Vinegar, - - - | 30 grammes. |
| | Honey, - - - - - - - - | 60 grammes. |
| | Barley Water, - - - - | 300 grammes. |
| | Yolk of Eggs, - - - - | q. s. |

Mix thoroughly, and divide into two lavements to be administered with an interval of an hour.—*Ibid.*

